# Reviewing a Decade of Change for Veterinarians: Past, Present and Gaps in Researching Stress, Coping and Mental Health Risks

**DOI:** 10.3390/ani12223199

**Published:** 2022-11-18

**Authors:** Birgit Ursula Stetina, Christine Krouzecky

**Affiliations:** 1 Faculty of Psychology, Sigmund Freud University Vienna, Freudplatz 1, 1020 Vienna, Austria; 2 Psychological Outpatient Clinic, Sigmund Freud University Vienna, Welthandelsplatz 3, 1020 Vienna, Austria

**Keywords:** veterinarians, stress, systematic review, suicide, bio-psycho-social model

## Abstract

**Simple Summary:**

Veterinarians work in a very complex profession; they operate within a triad (veterinarian, animal and animal handler) to take care of the animal. Stress and confrontations with a large variety of demands lead to a high risk for mental health problems. To gather additional insights on stressors we conducted a systematic review using several scientific databases indexing published studies of the last decade. The aim of the process was to structure stressors and burdens based on existing models to see potential gaps in research and find starting points for interventions. After employing a variety of exclusion criteria, 30 studies published in the last decade were included in the review and stressors were be grouped in biological, psychological and social stressors. Overall, most stressors were found in the social category which include interactions with animal handlers. This result indicates that communication with animal handlers is one of the main stressors in the veterinarian practice. Veterinarians need interventions that are tailored to their need to lower the risks for mental health disorders and ultimately suicide. With regards to the results more training of communication techniques might be an example for a starting point to decrease the burden for veterinarians. Although several dynamics of stressors, how they interact, is still unclear, research has found enough evidence to develop more specifically designed health promotion measures.

**Abstract:**

Veterinary medicine is a highly complex profession that includes a very specific set of stressors that range from individual to social aspects, with several of them being relevant risk factors for a variety of conditions. The aim of this systematic review was to identify and cluster the material on stressors and suicidality in the veterinarian practice published during the last 10 years. The systematic review was conducted employing the PRISMA (Preferred Reporting Items for Systematic Reviews and Meta-Analyses) guidelines using PubMed, PsycNet, Google Scholar, Medline, PsycINFO, PSYNDEX and Web of Science (2012–present) by two independent researchers resulting in the inclusion of 30 quantitative and mixed methods studies. Results of these studies on stressors were categorized using the bio-psycho-social model showing that social stressors play a prominent role. This category includes the largest number of stressors indicating that the human–human interactions in the veterinarian practice are the main stressor, underlining that training in communication techniques is a potential starting point for interventions. In addition to stressors, the results showed an additional category “psychological consequences” describing mental health disorders and suicide. Although there are still gaps in research there is enough evidence to establish more tailored health promotion measures for veterinarians.

## 1. Introduction

Veterinary medicine is a highly complex profession as veterinarians work within a triad consisting of the veterinarian, the animal and the animal handler. Therefore, veterinarians have responsibilities towards the animal, animal handlers/owners and regarding animal welfare in general. Daily work is based on a fundamental ethical dilemma, even described as the “fundamental problem in veterinary medicine” [1]: the person who gives consent to any form of treatment is not the patient who is undergoing that treatment, as Gray [2] describes. Rollin [3,4] points out that veterinarians have additional obligations to several other stakeholders as well as peers of the profession, society in general, themselves and potential employees. Given the large number of parties involved and the relevance of some of the topics (e.g., euthanasia, animal welfare concerns, changing medical environment and feminization), the interests of the stakeholders often come into conflict with each other. In combination with that the inner personal role conflict as main threat for our self-perception has to be pointed out [5]. Results of these conflicts are situations where it is unclear which path is best to take. The frequency of those situations is quite high. Batchelor and McKeegan [6] report that 57% of veterinarians in a UK sample of 58 practicing veterinary surgeons experience one to two dilemmas per week. More than half of the surveyed veterinarian surgeons in a UK sample report one to two ethical dilemmas per week and one third report three to five per week. From a psychological viewpoint the high number of ethical dilemmas is one piece of a uniquely complex job that includes a very specific set of stressors and burdens that range from individual aspects to social aspects with several of them being relevant risk factors for a variety of disorders and problems. This specific set seems to lead to a typical profile regarding stress management strategies in veterinarians including several maladaptive strategies [7]. The following examples illustrate the specific set of stressors and burdens.

Euthanasia is known as a main burden from a theoretical perspective [4] and is mentioned by veterinarians as one of the top stressors [6,7,8]. It needs to be seen as more than just the technical act. In addition to the burden to take an individual’s life it is the ambivalence that lies within the procedure that adds problematic aspects [9]. Arnold Arluke described the so-called caring–killing paradox in the context of animal shelter work in the words of an experienced shelter worker that the job includes “[…] to care for the animals, but also might have to kill some of them […]”. Psychologists might add that euthanasia as part of a job description means unpredictable emotional turmoil as part of the job. In combination with lack of support the highly relevant stressor seems obvious.

In addition to their own emotions, veterinarians as part of that complicated triad between themselves, the animal and the animal handler, are confronted with others’ emotions without sufficient training to contain or even manage them as part of a counseling process. To continue with the example of euthanasia, pet loss is the phenomenon that is experienced from the animal handler’s viewpoint. Authors describe the veterinarian’s role in pet loss [10] pointing out that in their study with animal handlers 34% state that they had the possibility to talk to their veterinarian after euthanasia. Based on that number it seems that there is a high number of animal handlers that need and obtain counseling from an emotionally strained person in a very complex situation. The emotional status of the veterinarian in that situation is not mentioned; it needs to be added to the unique set of stressors in the veterinarian practice.

Animal welfare is one of the main responsibilities in the veterinarian practice and is sometimes perceived differently by professionals and animal handlers, in some cases the animal handler is even a threat to the animal’s wellbeing. In small animal practice in particular, decision-making processes about what is best for the animal are influenced by contextual factors related to the client, the veterinarian, professional colleagues and the work environment [11]. Animal cruelty in different forms can be summarized from a psychological perspective as pathological forms of human–animal interactions. Veterinarians also treat abused animals and have to take care of the animal’s well-being. The confrontation with this dark side of the human–animal relationship is emotionally draining [12]. In addition, veterinarians experience an additional burden, because although they might suspect cruelty the sources can be unclear. In addition to that, some forms of abuse are still tabooed (even in research), therefore it is a difficult endeavor to recognize the signs.

The field of veterinary medicine is changing. With the establishment of specific professional associations in the late 20th century, the rise in small animal medicine intensified, as did the growth of clinical specialties and the demand from various markets such as pet food and insurance [13]. In addition, the increased demand for companion animals has led to an increase in the number of corporate veterinary clinics [14] which nevertheless, ended in a growing shortage reported in many countries: the supply is getting lower than the demand and veterinarians who leave the field add to the shortage. The growing shortage is not new, but the pure numbers are not the only aspect that needs mentioning [15]. Certain fields seem to be getting more attractive (small animal practice and specialized practices) and location is getting more and more relevant, most practitioners want to work in an urban area [16]. A total of 78.5% out of 28 European countries already experience a shortage in rural areas. The report points out that although not yet significant for the missing 21.5% evidence suggests that a shortage will happen. An analysis of US veterinarian changes shows that veterinarian “want to work lesser hours” and especially point out that the pandemic hurt veterinarian practices. The authors describe a decline in productivity of nearly 25% in 2020. Although numbers of appointments increased significantly since then it is made clear that the lack of stability in flow of patients, productivity and their own health adds to feeling overworked [17]. The stressors of a potential shortage include potentially getting a job in a less attractive region, needing to specialize in another field in veterinary medicine, and living and working under potentially unstable conditions; even more since the pandemic.

Further changes in the field come from a feminization, since most graduates are now females [7]. Student numbers for the academic year 2020–2021 show in Austria, for example, about 80.2% of veterinary students are female [18]. A total of 76.1% of graduates are female in Austria [18] which is also consistent with data from other countries such the United States, where the annual report of the American Association of Veterinary Medical Colleges (AAVMC) shows that the gender identity of more than 80 of the applicants for class 2025 is female [19]. The change during the last decades led to the current phenomenon that female practitioners are younger on average than their male colleagues. The set of stressors and burdens is different for female veterinarians, analysis show that in addition to discrimination in interactions the gender pay gap plays a relevant role as in almost all other occupations. Studies analyzing gender differences indicate differences in perception of workload and social support as published in a recent systematic review [20]. Female veterinarians seem to have specific stress management strategies and indicate a higher vulnerability for females with regards to their coping strategies [21]. Results of a systematic review from 2010 [22] show that female veterinarians are most at risk for negative stress outcomes such as suicidal ideation, mental health problems and job dissatisfaction. The mentioned stressors that should be added to the set are increasing responsibilities in the practice together with increasing family responsibilities, which are often assumed by female veterinarians. 

More intervention strategies and health promotion measures are needed. The combination of a lack of psychological support [23] and a stressful veterinarian practice leads to a high prevalence of risk factors such as suicide or depression [8]. Moreover, deficit coping strategies, which also may be related to the complex nature of the job, enhance the vulnerability of veterinarians for stress-related illnesses (e.g., burnout). Therefore, the concept of resilience becomes increasingly important. Instead of trying to intervene at a timepoint when veterinarians are already suffering measures should focus on protective health factors. Resilience might offer a promising construct as mental and physical health emerged as strong predictors of resilience among veterinarians [24]. 

While research is still trying to better understand how the dynamic of the described complexity in the line of veterinary work can be analyzed, suicide numbers in total are rising, and an increasing number of veterinarians are suffering from stress-related disorders. It seems one can observe a whole profession drifting towards a crisis without targeted prevention programs and major changes in public health measures. Therefore, the goal of this systematic review is to gather information to add a piece to the puzzle that explains what it means to work as a veterinarian from a bio-psycho-social viewpoint. The review is conducted to identify and collect the published material on changes, stressors and suicidality in the field of veterinary work to get closer to a general understanding of the mentioned dynamic of the complexity. During this process the main objective for the field is the identification of gaps in research and well-researched concepts with the intent to use those as starting points for health promotion measures. 

## 2. Materials and Methods 

The purpose of this systematic review is to provide an overview of studies published over the past decade on work-related stressors as well as associated effects in veterinarians. It was conducted according to the Preferred Reporting Items for Systematic Reviews and Meta-Analysis (PRISMA) guidelines [25,26]. Study procedures were specified in advance in a study protocol that described the search strategy, inclusion and exclusion criteria and data extraction.

### 2.1. Eligibility Criteria

The following eligibility criteria were used to select appropriate studies for review: (a) publication in English, (b) collection of empirical data on work-related stressors, stress and associated effects such as suicide or burnout (and connected synonyms) in veterinarian professionals, (c) report findings regarding existing stressors and suicide rates among currently practicing veterinarians and (d) articles published between 2012 and September 2022. 

Literature reviews, conference contributions and books were excluded. 

### 2.2. Search Procedure 

Studies were identified by searching the electronic databases PubMed, PsycNet, Google Scholar, Medline, PsycINFO, PSYNDEX and Web of Science (2012–present) by two independent researchers (B.U.S. and C.K.). The search terms were as follows: “veterinarians” OR “vets” OR “veterinary professionals” OR “veterinary surgeon” OR “veterinary technician” OR “practicing veterinarians” OR “veterinary physician” OR “veterinary practice” AND “stress” AND “mental stress” OR “psychological stress” OR “distress” OR “workload” OR “burden” OR “pressure” OR “tension” OR “tensity” OR “stress perception” OR “physiological stress” OR “mental work load” OR “burnout” OR “work stress” OR “suicide” OR “suicidal ideation” OR “suicidal tendency” OR “suicide risk factors” OR “suicidal behavior” OR “suicide warning signs” OR “suicidal”. 

The literature search resulted in 16,054 records which were transferred and documented in the reference management tool EndNote. Duplicates were manually excluded, and the remaining literature was screened by titles and abstracts independently by the two researchers (B.U.S and C.K.). Citations not matching the eligibility criteria were excluded. Afterward, remaining studies were reviewed in full-text and categorized in the three categories “suitable”, “other on topic” and “not suitable”. Investigators then extracted information on authors, year of publication, location, subject, study type/design, sample size and main findings of studies within the category “suitable”. Finally, researchers (B.U.S. and C.K.) assigned the extracted studies to the Oxford Center for Evidence-Based Medicine, to considerate the quality of the studies included in the review. 

## 3. Results

**Figure 1:**
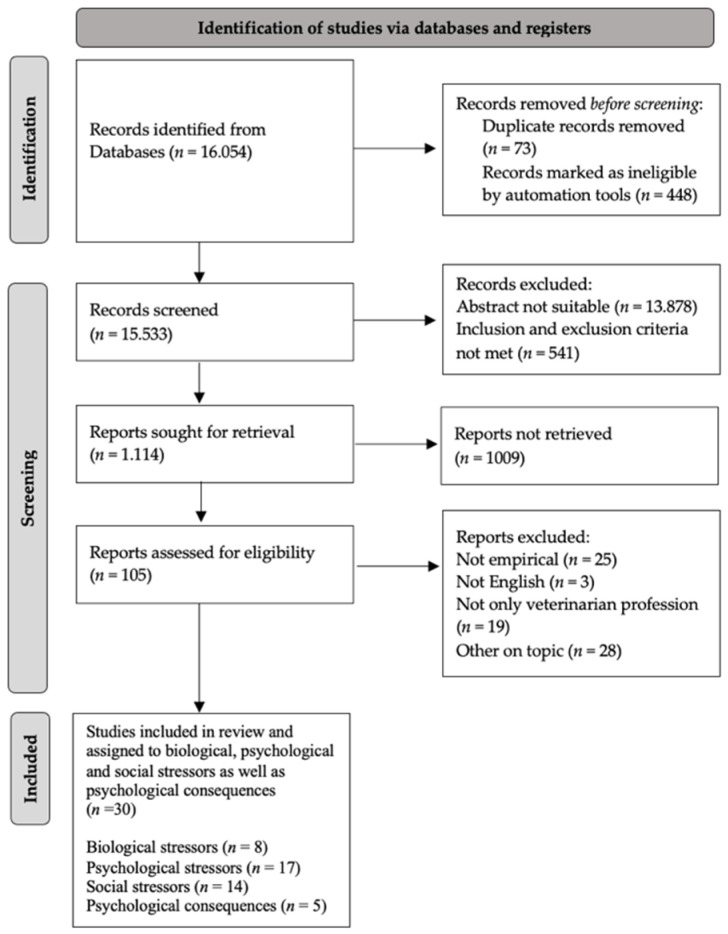
Flowchart.

The literature search resulted in 16,054 citations. After excluding duplicates, records marked as ineligible by automation tools and studies not matching the inclusion criteria (publication in English; collection of empirical data on work-related stressors, stress and associated effects such as suicide or burnout (and connected synonyms) in veterinarian professionals; report findings regarding existing stressors and suicide rates among currently practicing veterinarians and articles published between 2012 and September 2022), the final selection resulted in 30 sources (see Figure 1). This selection was assigned to biological (*n* = 8), psychological (*n* = 17) and social stressors (*n* = 14) referring to the interconnection between biological, psychological and social–environmental factors in the context of human health (=bio-psycho-social model) [27]. In addition, a category dealing with psychological consequences of veterinary practice (*n* = 5) was added because some studies did not address stressors but rather the prevalence of mental disorders within this professional group. Overall, there was a total of 44 assignments in 30 studies, as some studies could be allocated to several categories.

### 3.1. Biological Stressors

According to the bio-psycho-social model mentioned above, biological stressors include, for example, demographic factors such as biological sex or age. Eight studies dealt with such stressors, which might affect the field of veterinary practice (Table 1). In six of these eight studies, results suggested that female sex may have an impact on the mental health of veterinarians. In this context, it was found that female veterinarians are significantly more stressed by their work than the general population, but also than their male colleagues [7,20,28,29] This is shown by higher values of psychological distress (such as depression or anxiety), a higher level of burnout and negative attitudes toward social support [7,20,28,29]. Influencing factors seem to be longer working hours [29], personal problems [30], higher job demands and lower professional and personal resources compared to their male veterinarian counterparts [31]. In addition, one study showed that female veterinarians appear to apply dysfunctional stress processing strategies, which are more pronounced in female veterinarians than in male colleagues [6]. Besides these factors one study demonstrated that female veterinarians are more likely to seek professional help when they feel psychologically distressed [30] which might explain results of another study in this category which found that the proportional mortality rate for suicide among veterinarians appear to be significantly higher in male veterinarians [32]. The same study also found that the suicide rate among veterinarians is significantly higher (*p* < 0.001) when compared with the general population. In this context, data additionally suggested that an age of under 65 years seems to enhance the risk of suicidality in veterinarians [32].

### 3.2. Psychological Stressors

Stressors in this category refer to psychological factors that might have a negative impact on humans’ mental health or emotional well-being. Seventeen studies that addressed psychological stressors as possible factors influencing veterinarians’ work and related mental health were assigned to this category (Table 2). Most of these studies addressed various ethical dilemmas in veterinary practice that potentially impact mental health and suicide rates among veterinarians. In this context, studies demonstrated that veterinarians face at least one to two ethical challenges per week [5,16,30]. The most ethically and morally challenging situations identified in a large number of studies, were suspected pet abuse [31], interactions with animal caregivers [6,18], especially when there are financial constraints of them that prevent the implementation of the necessary treatment [31,32,33], and euthanasia [6,18,30,31,34,35,36]. Overall, studies demonstrate that ethical stressors can be associated with an increase in psychological distress such as anxiety, depression, burnout and suicide risk [6,16,18,30,31,34,35,36]. In addition to ethical challenges, several studies evaluated coping strategies in veterinarians which might be associated with the psychological well-being among veterinarians. Furthermore, studies found that veterinarians tend to use negative stress management strategies such as emotional avoidance strategies, alcohol abuse [36] and rumination [6] which might affect mental health in veterinarians. Some studies in this category also evaluated specific psychological factors in the context of mental health such as the personality of veterinarians as possible predictor of occupational stress; fearlessness about death and its association with euthanasia distress; and the COVID-19 depopulation event and its possible effect on mental health in swine veterinarians. Results demonstrated that most of these aspects significantly influence the psychological well-being in humans. With regard to the personality of veterinarians, for example, findings indicate that personality is a better predictor of occupational stress than environment and that neuroticism in particular is the trait that significantly predicts occupational stress [37]. Regarding the COVID-19 depopulation event and its possible effect on mental health among swine veterinarians’ findings showed that veterinarians’ involvement in this event is significantly associated with burnout and psychological distress [38]. The only stressor studied that did not appear to have an impact was fearlessness about death which appeared to not be a significant risk factor for psychological distress and suicidal behavior among veterinarians [34].

### 3.3. Social and Environmental Stressors

Social and environmental stressors are interpersonal factors such as social interactions and community activities as well as environmental factors such as workload and employment relationships. Fourteen studies were assigned to this category with most of them dealing with specific work-related stressors such as workload, financial issues, contact with clients or work–life balance (Table 3). In summary, the studies within this category demonstrated that issues with clients is the most frequently stated stressor negatively affecting veterinarian’s health [6,34,35,36,39,40,41,42,43]. According to one study this aspect is also linked to client satisfaction indicating that a higher client satisfaction might be associated with a lower mental health status among veterinarians [20]. This stressor of issues with clients is followed by the stressor of working hours and workload affecting the psychological well-being among veterinarians [6,22,34,36,44,45,46]. Findings in this context indicate that working overtime and an associated missing work–life balance especially in small animal veterinarians are significantly linked with higher levels of burnout and suicidal ideation [45,46].

### 3.4. Psychological Consequences

Five studies could not be classified as bio-psycho-social stressors because they addressed the psychological consequences of veterinary practice (Table 4). In this context, the prevalence of mental illnesses and suicidality have been investigated. Studies with regard to this topic, demonstrated that approximately one third of veterinarians experience depressive episodes and more than one fourth experience suicidal ideation [47]. In addition, findings showed that veterinarians achieve significantly higher scores regarding the level of burnout, compassion fatigue, anxiety and depression as well as significantly lower mean resilience when compared with the general population [24,48]. A longitudinal study further demonstrated that psychological distress and suicidal ideation increased when veterinarians are dissatisfied with their profession [49]. The consequence of this—namely that veterinarians reduce their working hours or stop working altogether, e.g., due to burnout—has also been investigated as an economic burden since the cost of employee turnover is almost twice as high as the cost of lost productivity due to reduced working hours [50].

### 3.5. Limitations

Stress and burden for veterinary practitioners have many sources. Several common mental health disorders could be included in the search terms as presented in other systematic reviews (20). In addition to the fact that there is a recent publication presenting the outcome of such a design the combination of including all methodologies, if described, and a larger variety of topics would lead to a broader but potentially less precise outcome. In the presented systematic review, quantitative and mixed methods design studies were included, the criterion “description of a method” was used without specifying which one. The used instrument for quality assessment of studies [51] allows to rate the quality of quantitative studies, but not qualitative studies. Therefore, the authors analyzed the quantitative parts in the mixed methods designs. As this might be seen as problematic, it was the planned course of action to include quantitative research and mixed methods approaches for comprehensive findings that cover aspects of the problem that might be hidden in solely quantitative approaches.

## 4. Discussion

The complexity and broadband of the topic is obvious being confronted with the variety of papers that fit the described criteria. The bio-psycho-social model of health seems like a very useful framework to categorize the published findings of the last decade. In addition to the affected areas of life another categorization might help to structure the potential needs of the field in an etiological way similar to a case formulation in cognitive behavioral therapy. Using that model we can structure our bio-psycho-social stressors and burdens into causal factors and vulnerabilities, triggers and precipitating factors and maintaining factors in an attempt to clarify the complex interaction of possible influences within the veterinary practice based on measurable constructs. Considering this in the context of the present study, the high suicide rate among veterinarians can also be understood as a result of these vulnerabilities, triggers and precipitating factors and maintaining factors. Based on the results presented, this would mean that we can hypothesize that suicide risk among veterinarians may be influenced by the fact that individuals entering the profession have specific characteristics that involve an individual predisposition or vulnerability [8]. This is also indicated by one of the studies included in the systematic review, which shows that individual factors such as personality traits are better predictors of occupational stress than are environmental factors [40]. The combination of individual vulnerability and confrontation with psychosocial stressors explained within the present study then clarifies the increased psychological distress among veterinarians, which the studies found to be more pronounced than that of the general population [7,32]. In addition, studies suggest that the practice of euthanasia in animals and suicide awareness in the industry are changing attitudes toward suicide. In this context, findings of a German study indicate that lower euthanasia levels can be associated with a more pronounced fearlessness about death (FAD) rate, even though an increased FAD was not identified as a significant risk factor for suicidal behavior [38]. Besides this aspect, veterinarians have direct access to and knowledge of means of suicide, which might affect planning phases that normally precede the actual suicidal act and hinders timely professional help (presuicidal syndrome) [52]. Regarding maintaining factors, the above-mentioned category of psychological consequences indicates that psychological distress and suicidal ideation among veterinarians has increased within the last decade and although the biopsychosocial stressors are already visible, no action is being taken to reduce them [49,50]. This circumstance will inevitably lead to an increasing burden on veterinarians and the associated increase in the suicide rate which is why this aspect can be seen as a maintaining factor. Although this additional categorization does not provide a solution for the main problem presented, it is useful in highlighting the complex interactions of possible mechanisms over the course of a veterinary practice, allowing for a more systematic approach to research that can serve as a basis for long-term change.

Interestingly, changes in the profession as mentioned earlier are not a core topic in the results on researching stressors as described in the methods section. Though the results show that the main stressor can be found thinking about the dynamics of the so-called “fundamental problem of veterinary medicine” [1] and lies within the interactions with the animal handlers, communication issues and overly controlling or unrealistic animal handlers are the mentioned stressors. Both aspects can be condensed to the topic of communication. Perceived lack of communication skills makes it difficult to interact and to share relevant information which leads to subjective insecurities in the interaction and creates tension. Veterinarians need more communication skills and techniques in their daily work, but these skills are only covered rudimentarily in their education. While there is a need to perform sensible forms of communication, especially with animal handlers, it is not possible to use certain communication skills without being trained (cognitive level), thus leading to a daily experience of cognitive dissonance [53]. Although there are individuals who adopt communication skills quickly and seem very natural in their interactions it is still a stressor to need to perform in an area that one is not trained in sufficiently. Perret and colleagues [24] even show indirectly that this inner struggle is not necessarily visible to the animal handler, but the result might be that the animal handler’s satisfaction is high, and the veterinarian’s burden is high, leading to a lower mental health status in veterinarians. In addition to that it needs to be mentioned that communication skills are not the only skills that support adaptive problem-solving and healthy coping strategies. As mentioned before a very specific skillset is needed for an adaptive stress management. Many new curricula and studies are published, mostly US, describing more or less revolutionary ways to include these contents in students training and several US veterinary schools already focus on communication as regularly presented by the American Association of Veterinary Medical Colleges [54]. The daily hassles and stressors might be too much, even if all skills are present and used in the correct way. There will always be interactions, decisions and a lot more that should be reflected with a neutral professional to stay healthy. For this reason, the authors promote counseling as health promotion measure for veterinarians.

Several stressors that can be condensed under communication issues seem to have gained relevance during the last decade. Especially problematic situations are where specific communication techniques might help, and the veterinarian is somehow under pressure because the animal handler’s ideas and demands either risk animal welfare, are potentially abusive or are unrealistic. Examples of unrealistic expectations could be very specific ideas about the treatment of their animals that are outside of the veterinarian’s expertise or even outside of the common practice in an area or country. Health related information can be found online for humans and animals, clients “Google” symptoms, as it is commonly referred to. At least three quarters of adults use the internet for health-related information regarding human health [55] and a similar number (76.4%) is reported for pet health information in a US sample [56]. Whereas, the results from a UK sample show that for 78.6% of information seekers the Internet is the most frequently used information source [57]. This practice can be highly problematic, because it is difficult to assess the quality of the gathered information, there are no regulations for health-related information online (although concepts existed) and a differential diagnostic process cannot be replaced by an online search engine. However, online information can be empowering as well, and well-researched information can have a supportive effect in difficult situations as well as online (support) groups [58]. However, for the professional on the other side this common practice of searching for information online is a relevant stressor. In human health professionals it was found, during the early years of health-related online research, that the used communication strategy was mostly deflection and not to allow a discussion about online information. For veterinarians, a UK study shows that more than half of the surveyed professionals report a negative impact on the professional relationship [59]; an earlier study even mentions a negative impact on pet health [60]. Researchers [61] pointed out that the potential reaction to an “Internet informed” individual in addition to a defensive strategy (professional centered approach), one could collaborate and obtain information together (client centered approach) or even go so far as to guide them to reliable information online (Internet prescription). The acknowledgement of the search for knowledge was mentioned as a key component for a successful communication strategy for veterinarians as well [58]. Veterinarians from a European sample (AT, DK and UK) seem to be well aware that a large group of animal handlers searches online for information [62]. Around three quarters of them report that their professional advice is occasionally questioned by animal handlers [62], which is especially interesting taking into consideration that most pet owners from another UK sample mention that they discuss online information with their veterinarian “sometimes” [57]. 

To summarize, there are several starting points for interventions such as communication strategies for the “Internet informed” animal handler. There is a strong need for tailored interventions for veterinarians. Health promotion measures ranging from information campaigns to preventive measures to normalize mental health topics are needed. Training in psychosocial skills including communication as well as adaptive stress management strategies can only be the beginning of services to help veterinarians cope with stress and ultimately reduce the suicide rate. In addition, there are several knowledge gaps and open questions that might help to design better measures and direct future research. What additional learnings could the field generate from analyzing gender differences in seeking professional help? What is the subjective experience of the changes in the veterinary medical profession?

## Figures and Tables

**Table 1:** Biological stressors.

First Author, Year of Publication and Location	Subject	OCEBM Level (2011)	Study Type/Design	Main Findings
Dalum et al. (2022); Norway [30]	Investigation of the self-reported prevalence of suicidal thoughts and behaviors and contributing and independent factors associated with suicidal thoughts and behaviors among veterinarians in Norway	IV	cross-sectional survey	An online survey was conducted to investigate the prevalence of suicidal thoughts and behavior among veterinarians in Norway and possible gender differences. In addition, the study tried to examine individual and work-related stressors associated with suicidal thoughts. The questionnaire included demographic data and the following standardized measurements: the Paykel Suicide Scale, the Basic Character Inventory, the SCL-5 and the Job Stress Questionnaire. In total, 2596 participants were included in statistical analyses (69% female and 31% male). The results demonstrated, that 27% of veterinarians in Norway felt that life was not worth living during the last year, 5% had serious suicidal thoughts and 0.2% had attempted suicide. Female veterinarians reported a significantly higher prevalence of suicidal feelings and thoughts than their male colleagues. Severe suicidal ideation occurred almost twice as frequently in females as in their male colleagues (6.2% vs. 3.6%, χ2 = 6.5, *p* = 0.011). In addition, statistical analyses showed that being single (OR = 1.76; 95% CI = 1.13–2.72; *p* < 0.05), negative life events (OR = 2.75; 95% CI = 1.22–1.68; *p* < 0.001) and the presence of mental distress (OR = 2.75; 95% CI = 2.14–3.52; *p* < 0.001) were independent factors associated with serious suicidal thoughts. On a personal level, veterinarians (especially females) related their serious suicidal thoughts to work (48.1%) and personal problems (36.8%) and a lesser degree to family (20.8%), social (18.8%) and other problems (27.4%).
Dalum et al. (2022); Norway [33]	Investigation of the prevalence of mental health problems and professional help-seeking behavior for such problems, and the independent factors associated with help-seeking behavior among veterinarians in Norway	IV	cross-sectional survey	An online survey was conducted to evaluate the prevalence of self-reported mental health problems in need of treatment among veterinarians in Norway as well as the number of veterinarians who have actually sought professional help. In addition, the study wanted to find out the veterinarians’ opinion on factors contributing to their mental-health problems and factors associated with professional-help seeking. The questionnaire included demographic data and the following standardized measurements: the Paykel Suicide Scale, the Basic Character Inventory, the SCL-5 and the Job Stress Questionnaire. In addition, questions regarding mental health problems in need of treatment were asked. The same sample of 2596 veterinarians as the 2022 study regarding suicidal thoughts were included in statistical analyses (70% women, 30% men). Results demonstrated that 30% (95% CI 28.1–31.8%) of the sample reported mental health problems in need of treatment with females reporting significantly more problems in this context (36% vs. 15%; χ2 = 107.9, *p* < 0.001). Moreover, findings indicate that 54% had sought professional help, women significantly more often (56%) than men (41%) (χ2 = 9.06, *p* = 0.003). Veterinarians most often associated mental health problems with work problems (47%), and women significantly more often (49%) than men (34%) (χ2 = 8.2, *p* = 0.004). Factors significantly related to help-seeking were being female (OR = 2.11 (95% CI: 1.24–3.60)), working with production animals (OR = 0.35 (0.13–0.98)), working in public administration (OR = 2.27 (1.15–4.45)), science/research (OR = 4.78 (1.99–11.47)), or “other” fields (OR = 2.79 (1.23–6.32)) and attitudes toward mental illness (OR = 1.32 (1.03–1.68)).
Emmett et al. (2019); Austria, Germany [7]	Identification of a profile of typical coping strategies in female veterinarians	IV	cross-sectional survey	An online survey was conducted to investigate typical stress management strategies in female veterinarians in Austria and Germany. The questionnaire included demographic data and the standardized measurement Stressverarbeitungsfragebogen (SVF-120). In total, data of 78 female veterinarians were included in statistical analyses. Results demonstrated that female veterinarians are significantly more likely to use negative stress management methods such as rumination (t(74) = 6.733; *p* = < 0.001; d = 0.726) or escape (t(72) = 2.173; *p* = 0.033; d = 0.281) compared to the normal population. In addition, specific job stressors were identified, such as communication with animal caregivers (54.3%), euthanasia (11.4%) and emergency services with 24/7 availability (10%).
Kassem et al. (2019); U.S. [28]	Analyzing attitudes toward mental illness among U.S. veterinarians	IV	cross-sectional survey	A questionnaire was developed to analyze characteristics (demographic, occupational and mental health) associated with negative attitudes toward mental illness among U.S. veterinarians. The questionnaire included demographic data, the Kessler Psychological Distress Scale and questions regarding suicidal ideation, attempted suicide, depression, treatment effectiveness and social support. A total of 9522 data sets were included in statistical analyses (69.2% female and 30.8% male). Results demonstrated that 47.3% of the sample had a negative attitude toward social support and 3.1% had a negative attitude toward treatment effectiveness. Specific demographic characteristics favored the negative attitude toward treatment effectiveness (male (40.9%), solo practitioners (27.2%), with evidence of serious psychological distress (22%) and those reporting suicidal ideation after graduating from veterinary school. Typical characteristics for a negative attitude toward social support were: female (75.2%), childless (46.8%), unmarried (26.5%), 40–50 years old (47.3%), solo practitioners (71.1%), with evidence of serious psychological distress, those reporting depression after graduating from veterinary school (24.1%).
Mastenbroek et al. (2014); The Netherlands [31]	Examining burnout, work engagement, and gender differences among veterinarians who graduated in the past 10 years	IV	cross-sectional survey	A self-report questionnaire was conducted to analyze levels of burnout and work engagement as well as characteristics predicting burnout and work engagement. The questionnaire included demographic data and the following standardized questionnaires: the Utrecht Work Engagement Scale, the Maslach Burnout Inventory-General Survey and the Veterinary Demands and Resources Questionnaire. In total, 860 data sets were included in the statistical analyses (73% females and 27% males; mean age = 32 years, SD = 4.4). Results overall demonstrated that the levels of exhaustion (M = 1.66, SD = 1.14), cynicism (M = 1.18, SD = 1.10) and work engagement (M = 3.63, SD = 1.05) were significantly lower among veterinarians compared to the norm group (d = 0.1 for all three measures). Moreover, data indicated that male veterinarians were less exhausted (B = −0.24, Beta = −0.09) and more engaged (B = 0.23, Beta = 0.10) than female veterinarians. In addition, results showed that female veterinarians experience slightly higher job demands and moderately lower professional and personal resources compared to their male counterparts. Furthermore, results suggest that variance in exhaustion and cynicism is best explained by job demands (Exhaustion: R2 (male) = 0.52, R2 (female) = 0.49; Cynicism: R2 (male) = 0.49, R2 (female) = 0.40) and job resources (Exhaustion: R2 (male) = 0.38; R2 (female) = 0.33; Cynicism: R2 (male) = 0.56; R2 (female) = 0.33) and to a lesser extent by personal resources (Exhaustion: R2 (male) = 0.21; R2 (female) = 0.19; Cynicism: R2 (male) = 0.10; R2 (female) = 0.19). Variance in work engagement may be best explained by job (R2 (male) = 0.44; R2 (female) = 0.48) and personal resources (R2 (male) = 0.42, R2 (female) = 0.31).
Pohl et al. (2022); Germany [20]	Investigation of work-related stressors and their effects on mental health among veterinarians	II	Systematic review	A systematic review was performed to provide an overview of existing work-related stressors and their effects on mental health among veterinarians. Therefore, 21 studies related to work-related stressors, psychological workload and potential effects such as burnout or depression were analyzed. Results demonstrated a high prevalence of psychological stressors in veterinary practice. Studies in this context indicated that the risk of burnout, anxiety and depressive disorders are higher among veterinarians than in the general population. In addition, the results showed that working hours and ethical dilemmas are the main sources of stress perceived as more stressful by female veterinarians than by their male colleagues.
Shirangi et al. (2013); Australia [29]	Investigation of mental health in female veterinarians and possible influencing factors (demographic and work-related)	IV	cross-sectional survey	An online survey was conducted to investigate the overall mental health status in female veterinarians, to evaluate demographic and work-related characteristics as possible influencing factors on mental health and to determine differences in terms of working hours in female veterinarians with children and without children. The questionnaire included demographic data and the following standardized measurements: the General Health Questionnaire, the Affective Well-Being Scale, the Positive and Negative Affect Scale and the Social Support at Home instrument. In total, 1017 data sets of female veterinarians (mean age = 35.1 years) were included in statistical analysis. Results demonstrated that 37% of respondents were suffering from minor psychological distress by the time of the survey. In addition, findings suggest that women with two or more children had less anxiety (M = 3.97, SD = 0.83) and depression (M = 4.53, SD = 0.73) compared with those who had never been pregnant or were childless (M = 3.6, SD = 0.83 for anxiety; M = 4.13, SD = 0.8 for depression). Moreover, results showed that longer working hours were associated with increased anxiety (M = 3.5, SD = 0.9) and depression (M = 4.2, SD = 0.9) among female veterinarians.
Tomasi et al. (2019); U.S. [32]	Assessment of proportional mortality rates (PMRs) for suicide among female and male U.S. veterinarians from 1979 to 2015	II	Systematic review	To examine the proportional mortality rate (PMRs) for suicide among veterinarians in the context of demographics, life insurance databases and AVMA obituaries were systematically searched. In a total of 398 death certificates of 11,629 deceased, the cause of death was reported as suicide (82% of men and 18% of women). Most were under 65 years of age. The PMRs for suicide for all veterinarian decedents (males: 2.1 (95% CI = 1.87–2.32); females: 3.5 (95% CI = 2.73–4.39); *p* < 0.001) were significantly higher than for the general population. Overall, the numbers of suicide increased with each 5-year period.

**Table 2:** Psychological stressors.

First Author, Year of Publication and Location	Subject	OCEBM Level (2011)	Study Type/Design	Main Findings
Batchelor et al. (2012); UK [6]	Ethical challenges and associated stress faced by veterinarian professionals was investigated via questionnaire	IV	cross-sectional survey	A brief questionnaire was sent to veterinarians in the UK. The questionnaire included demographic data as well as typical ethical challenges (e.g., euthanasia of a healthy animal, client financial constraints and client desire to continue treatment despite compromised animal welfare) to be assessed according to the stress triggered on a scale between 0 to 10. The survey was completed by 58 veterinarians (43 females and 15 males). Fifty-seven percent of the participants stated to face at least one to two ethical challenges per week. The client’s desire to continue treatment despite compromised animal welfare was rated to be the most stressful challenge with a mean value of 9. Significant sex differences were found in stress ratings for healthy animal euthanasia (*p* = 0.02) and client wishing to continue treatment (*p* = 0.014) with females rating these challenges to be more stressful. The most common challenge faced was financial limitations restricting treatment options (55%).
Baysinger et al. (2022); U.S. [39]	Evaluation of the mental health of swine veterinarians involved in mass depopulations associated with COVID-19	IV	cross-sectional survey	An online survey was conducted to evaluate swine veterinarians’ experiences and perceptions regarding the COVID-19 depopulation event from April 2020 to June 2020. Besides demographic data, the following measurements were included in the questionnaire: the Cantril Self-Anchoring Scale, the Physician well-being Index, the burnout scale, The Kessler Psychological Distress Scale and the Brief Resilience Scale. A total of 134 veterinarians (34.1% female and 65.9% male) were included in the statistical analyses. Results demonstrated that depopulation involvement was significantly associated with burnout (F(132) = 12.41, *p* < 0.001). Additionally, data showed that the depopulation method utilized significantly impacted the evaluation of distress (F(76) = 7.63, *p* = 0.007), the perception of others (F(76) = 20.77, *p* < 0.001) and burnout F(132) = 17.02, (*p* < 0.001). Furthermore, 29% of the participants reported moderate levels of burnout.
Crane et al. (2015); Australia [35]	Investigation of morally challenging stressors in relation to psychological distress, perfectionism and resilience	IV	cross-sectional survey	An online survey was conducted of 540 Australian registered veterinarians (64.2% female; 35.8% male) aged 23 to 74 years (M = 41.06, SD = 11.53). The questionnaire included demographic variables and standardized measurements such as: the Multidimensional Perfectionism Scale, the Depression, Anxiety, Stress Scale, the Positive and Negative Affect Scale and the Brief Resilience Scale. Results indicated that suspected pet abuse was considered on average the most morally significant issue faced (M = 84.23, SD = 20.46). In addition, the data showed that working with clients who could not pay for recommended treatment was considered the least morally significant (M = 59.84, SD = 28.40) but most frequently endorsed stressor (n = 530), and that performing euthanasia was generally the most frequently occurring stressor (M = 7.41, SD = 1.54), nearly tied with the least morally significant stressor (M = 60.08, SD = 33.77). Statistical analyses moreover demonstrated that although morally significant stressors were associated with an increase in milder expressions of stress, they did not appear to be associated with more severe impairments in psychological well-being (*p* = 0.89). Rather, it was the combination of these triggering stressful events and trait perfectionism that appeared to create vulnerability to moral stressors (*p* = 0.97).
Dalum et al. (2022); Norway [30]	Investigation of the self-reported prevalence of suicidal thoughts and behaviors and contributing and independent factors associated with suicidal thoughts and behaviors among veterinarians in Norway	IV	cross-sectional survey	An online survey was conducted to investigate the prevalence of suicidal thoughts and behavior among veterinarians in Norway and possible gender differences. In addition, the study tried to examine individual and work-related stressors associated with suicidal thoughts. The questionnaire included demographic data and the following standardized measurements: the Paykel Suicide Scale, the Basic Character Inventory, the SCL-5 and the Job Stress Questionnaire. In total, 2596 participants were included in statistical analyses (69% female and 31% male). The results demonstrated that 27% of veterinarians in Norway felt that life was not worth living during the last year, 5% had serious suicidal thoughts, and 0.2% had attempted suicide. Female veterinarians reported a significantly higher prevalence of suicidal feelings and thoughts than their male colleagues. Severe suicidal ideation occurred almost twice as frequently in females as in their male colleagues (6.2% vs. 3.6%, χ2 = 6.5, *p* = 0.011). In addition, statistical analyses showed that being single (OR = 1.76; 95% CI = 1.13–2.72; *p* < 0.05), negative life events (OR = 2.75; 95% CI = 1.22–1.68; *p* < 0.001) and the presence of mental distress (OR = 2.75; 95% CI = 2.14–3.52; *p* < 0.001) were independent factors associated with serious suicidal thoughts. On a personal level, veterinarians (especially females) related their serious suicidal thoughts to work (48.1%) and personal problems (36.8%) and a lesser degree to family (20.8%), social (18.8%) and other problems (27.4%).
Dawson et al. (2017); UK [40]	Investigation of personality as predictor of occupational stress in veterinarians	IV	cross-sectional survey	An online survey was conducted to evaluate the personality of veterinarians as a possible predictor of occupational stress. The questionnaire included demographic data as well as the NEO Five-Factor Inventory, the Maslach Burnout Inventory and the Job Stress Survey. In total, 363 fully completed data sets were included in statistical analyses (220 female, 139 male, 4 others; mean age = 39 years, SD = 10.7). Results demonstrated that personality is a better predictor of occupational stress than environment (*p* < 0.001). Moreover, data showed that neuroticism is the trait that significantly predicts occupational stress (*p* < 0.001), and the components of neuroticism that contribute most to stress are depression (*p* = 0.002) and anger hostility (*p* = 0.005). Demographic factors, such as the number of years the veterinarian had been qualified, acted as a mediator between depression and occupational stress (*p* < 0.001), and as a moderator between personal achievements and occupational stress (*p* = 0.028).
Emmett et al. (2019); Austria, Germany [7]	Identification of a profile of typical coping strategies in female veterinarians	IV	cross-sectional survey	An online survey was conducted to investigate typical stress management strategies in female veterinarians in Austria and Germany. The questionnaire included demographic data and the standardized measurement Stressverarbeitungsfragebogen (SVF-120). In total, data of 78 female veterinarians were included in statistical analyses. Results demonstrated that female veterinarians are significantly more likely to use negative stress management methods such as rumination (t(74) = 6.733; *p* = < 0.001; d = 0.726) or escape (t(72) = 2.173; *p* = 0.033; d = 0.281) compared to the normal population. In addition, specific job stressors were identified, such as communication with animal caregivers (54.3%), euthanasia (11.4%) and emergency services with 24/7 availability (10%).
Glaesmer et al. (2021); Germany [38]	Investigation of German veterinarians regarding fearlessness about death (FAD) and its association with euthanasia distress	IV	cross-sectional survey	An online questionnaire was conducted to evaluate a possible connection between fearlessness about death (FAD) and euthanasia distress. The questionnaire included demographic data and the following standardized measurements: the Acquired Capability for Suicide Scale—Fearlessness About Death and the Euthanasia Distress Scale. Two samples of German veterinarians as well as a comparison group were recruited. Sample 1 (n = 3179 veterinarians, final sample = 3118 after exclusion; 97.5% female, mean age = 40.9, SD = 10.6) was recruited online and Sample 2 (n = 1963 comparison group; 55.3% female; mean age 46.3 years, SD = 13.4) was collected with the assistance of a demographic consulting company. Results demonstrated no significant differences concerning FAD between German veterinarians and the comparison sample (F = 0.968, *p* = 0.407). This indicates that an increased FAD is not a significant risk factor for suicidal behavior in veterinarians even though lower euthanasia levels (as an indicator of habituation to euthanasia) might be associated with higher levels of FAD (F = 3.177, *p* = 0.023).
Griek et al. (2018); U.S. [36]	Development of a comprehensive taxonomy of practice-related stressors experienced by US veterinarians	IV	cross-sectional survey	A questionnaire was designed to evaluate the prevalence of risk factors for suicide, attitudes toward mental illness and practice-related stressors among 11,627 U.S. veterinarians. The questionnaire included demographic data and questions regarding attitudes toward mental health, history of depression and mental health treatment, stressors related to veterinary medicine and satisfaction related to veterinary medicine. In addition, veterinarians had the opportunity to openly identify individual work-related stressors. A subset of 1422 US veterinarians provided written responses to the questionnaire. Based on quantitative and qualitative results, a model was developed with 15 categories of general practice-related stressors and 40 subcategories of more specific practice-related stressors. The most common practice-related stressors included financial insecurity (20.3%), problems with clients (17.9%), problems with staff or interpersonal issues (12.7%) and work–life balance (11.7%). The most common subcategories were clients unwilling to pay (8.3%), low income (6.9%), cost of maintaining practice (3.9%) and government or state agency policies (3.4%).
Kassem et al. (2019); U.S. [28]	Analyzing attitudes toward mental illness among U.S. veterinarians	IV	cross-sectional survey	A questionnaire was developed to analyze characteristics (demographic, occupational and mental health) associated with negative attitudes toward mental illness among U.S. veterinarians. The questionnaire included demographic data, the Kessler Psychological Distress Scale and questions regarding suicidal ideation, attempted suicide, depression, treatment effectiveness and social support. A total of 9522 data sets were included in statistical analyses (69.2% female and 30.8% male). Results demonstrated that 47.3% of the sample had a negative attitude toward social support and 3.1% had a negative attitude toward treatment effectiveness. Specific demographic characteristics favored the negative attitude toward treatment effectiveness (male (40.9%), solo practitioners (27.2%), with evidence of serious psychological distress (22%) and those reporting suicidal ideation after graduating from veterinary school. Typical characteristics for a negative attitude toward social support were: female (75.2%), childless (46.8%), unmarried (26.5%), 40–50 years old (47.3%), solo practitioners (71.1%), with evidence of serious psychological distress, those reporting depression after graduating from veterinary school (24.1%).
Kipperman et al. (2018); U.S. [34]	Investigation of U.S. veterinarian’s opinions regarding the frequency and nature of ethical dilemmas and associated aspects	IV	cross-sectional survey	An online questionnaire was conducted to investigate U.S. veterinarian’s opinions regarding the frequency and nature of ethical dilemmas encountered, beliefs regarding euthanasia and balancing client and animal interests, the prevalence and value of ethical training and suggestions for mitigating the stressful effects of ethical dilemmas. The questionnaire included demographic data and questions regarding clinical scenarios which were selected because they may be associated with ethical dilemmas, questions regarding euthanasia, questions about individual values in relation to ethical situations, questions regarding methods used to address ethical dilemmas and the impact of ethical dilemmas on work-related stress. In total, responses from 484 veterinarians were analyzed (80% female, 20% male; median age = 50 years). Results demonstrated that 52% of the sample experiences an ethical dilemma at least weekly. Scenarios involving client financial concerns were commonly reported causes of ethical conflicts. Most respondents (52%) indicated that ethical dilemmas were the main cause of work-related stress. Lower job experience and female gender were identified as characteristics for more strongly experienced stress regarding ethical dilemmas.
Mastenbroek et al. (2014); The Netherlands [31]	Examining burnout, work engagement and gender differences among veterinarians who graduated in the past 10 years	IV	cross-sectional survey	A self-report questionnaire was conducted to analyze levels of burnout and work engagement as well as characteristics predicting burnout and work engagement. The questionnaire included demographic data and the following standardized questionnaires: the Utrecht Work Engagement Scale, the Maslach Burnout Inventory-General Survey and the Veterinary Demands and Resources Questionnaire. In total, 860 data sets were included in the statistical analyses (73% females and 27% males; mean age = 32 years, SD = 4.4). Results overall demonstrated that the levels of exhaustion (M = 1.66, SD = 1.14), cynicism (M = 1.18, SD = 1.10) and work engagement (M = 3.63, SD = 1.05) were significantly lower among veterinarians compared to the norm group (d = 0.1 for all three measures). Moreover, data indicated that male veterinarians were less exhausted (B = −0.24, Beta = −0.09) and more engaged (B = 0.23, Beta = 0.10) than female veterinarians. In addition, results showed that female veterinarians experience slightly higher job demands and moderately lower professional and personal resources compared to their male counterparts. Furthermore, results suggest that variance in exhaustion and cynicism is best explained by job demands (Exhaustion: R2 (male) = 0.52, R2 (female) = 0.49; Cynicism: R2 (male) = 0.49, R2 (female) = 0.40) and job resources (Exhaustion: R2 (male) = 0.38; R2 (female) = 0.33; Cynicism: R2 (male) = 0.56; R2 (female) = 0.33) and to a lesser extent by personal resources (Exhaustion: R2 (male) = 0.21; R2 (female) = 0.19; Cynicism: R2 (male) = 0.10; R2 (female) = 0.19). Variance in work engagement may be best explained by job (R2 (male) = 0.44; R2 (female) = 0.48) and personal resources (R2 (male) = 0.42, R2 (female) = 0.31).
Moses et al. (2018); North America [37]	Analyzing ethical conflicts and moral distress in veterinarians	/	mixed-methods design	An online survey was conducted to analyze ethical conflicts and moral distress in North American veterinarians. The questionnaire included open and closed questions regarding the veterinarian’s perception of work-related ethical conflicts and associated moral distress. In total 889 fully completed data sets were included in quantitative and qualitative analyses. Results demonstrated that a majority of respondents reported conflicts about what care is appropriate to provide. Over 70% of respondents reported that the barriers they faced that prevented them from providing appropriate care caused them or their staff moderate to severe distress. Seventy-nine percent of participants reported being asked to provide care that they felt was pointless. More than 70% of participants reported not being trained in conflict resolution or self-care.
Platt et al. (2012); UK [22]	Obtaining information from veterinarians with suicidal ideation or behavior about factors associated with suicidality in their profession	/	mixed-methods design	To investigate work-related factors associated with suicidality among veterinarians 21 UK veterinarians (76% females, 24% males) who had attempted suicide or reported recent suicidal ideation were interviewed using semi-structured interview schedules. The interviews included topics relating to contributory factors, coping mechanisms and suicide prevention in the veterinary profession. In addition to the interviews, some topics were also assessed in part by fixed-response scales such as the Suicidal Intent Scale or items from the Motives for Parasuicide Questionnaire. Results demonstrated common factors contributing to suicidal behavior were workplace relationships, career concerns, problems with patients, number of hours and volume of work and responsibilities, although 2/3 of participants reported concurrent difficult life events. About half of the veterinarians had received a psychiatric diagnosis after their suicidal behavior. Participants suggested several possible preventive measures.
Platt et al. (2012); UK [41]	Investigation of suicidal behavior and psychosocial problems in veterinary profession	II	Systematic review	A systematic review was performed to summarize the knowledge on suicidal behavior and psychosocial problems in the veterinarian profession. Data from 52 studies (from 48 papers) concerning suicidal behavior, mental health problems, stress, burnout, occupational difficulties and psychosocial characteristics of veterinarians were analyzed. Results demonstrated that a majority of studies dealt with occupational stressors among veterinarians including managerial aspects of the job, long working hours, heavy workload, poor work–life balance, difficult client relations and performing euthanasia. In addition, some studies suggested that young and female veterinarians are at higher risk for negative outcomes such as suicidal thoughts, psychological problems and job dissatisfaction.
Pohl et al. (2022); Germany [20]	Investigation of work-related stressors and their effects on mental health among veterinarians	II	Systematic review	A systematic review was performed to provide an overview of existing work-related stressors and their effects on mental health among veterinarians. Therefore, 21 studies related to work-related stressors, psychological workload and potential effects such as burnout or depression were analyzed. Results demonstrated a high prevalence of psychological stressors in veterinary practice. Studies in this context indicated that the risk of burnout anxiety and depressive disorders are higher among veterinarians than in the general population. In addition, the results showed that working hours and ethical dilemmas are the main sources of stress perceived as more stressful by female veterinarians than by their male colleagues.
Tran et al. (2014); Australia [42]	Investigation of the association between euthanasia-administration frequency and mental health among veterinarians	IV	cross-sectional survey	Currently practicing Australian veterinarians were surveyed online using a cross-sectional design. The questionnaire included demographic data, work characteristics, questions relating to the euthanasia frequency and the following standardized measurements: the Depression, Anxiety, Stress Scale and the Suicide Behaviors Questionnaire–Revised. 540 data sets were included in statistical analyses (63.8% females, 36.2% males). Results showed that the administration of unauthorized euthanasia (i.e., euthanasia with which the veterinarian disagreed) was not related to the evaluated mental health variables (R2 = 0.005). In contrast, overall frequency of euthanasia had a weak positive linear relationship with depression (t = 2.55, *p* < 0.02; Beta = 0.13; Semipartial = 0.01). Moreover, the overall frequency of euthanasia moderated the effects of depression on suicide risk (Z = 8.46, *p* < 0.01; OR = 0.73). The type of this moderation suggested that the average frequency of euthanasia per week attenuates the association between depressed mood and suicide risk.
Wallace (2017); U.S. [43]	Evaluation of work-related stressors, burnout and suicidal ideations among veterinarians	IV	cross-sectional survey	An online questionnaire was conducted to investigate links between work-related stressors, burnout and suicidal ideation among U.S. veterinarians. In addition, individual coping strategies were explored. The questionnaire included demographic variables as well as the following standardized measurements: the National Survey of Psychiatric Morbidity and the Maslach Burnout Inventory. Additionally, questions regarding job demands, job control, social support and coping strategies were asked. In total, data sets of 464 veterinarians working in clinical practice were included in statistical analyses (31% females and 69% males; mean age = 43 years). Results demonstrated that burnout was associated with suicidal thoughts (Beta = 0.37) and appeared to be particularly important in understanding how unrealistic client demands indirectly contribute to suicidal ideation (Beta = 0.12). Furthermore, data indicated that job demands (especially work overload) (Beta = 0.25) are relevant indicators to burnout and that working conditions that are emotionally exhausting for veterinarians might foster suicidal ideation (work overload (r = −0.15), euthanasia (r = −0.40), animal suffering (r = −0.23) and unrealistic clients (r = −0.04). Results also showed that emotional avoidance strategies and alcohol abuse not only exacerbate burnout (Beta(avoidance) = 0.11; Beta(alcohol) = 0.12) and suicidal thoughts (Beta(avoidance) = 0.06; Beta(alcohol) = 0.13), but also increase the harmful effects of certain job demands (β = 0.13). Social support (Beta(Burnout) = −0.16; Beta(Suicide) = −0.13) and a supportive work environment (β = −0.13) has proven to be a relevant coping resource.

**Table 3:** Social and environmental stressors.

First Author, Year of Publication and Location	Subject	OCEBM Level (2011)	Study Type/Design	Main Findings
Andela, M. (2020); France [44]	Developing an instrument assessing the stressors met by veterinarians	IV	Systematic review + cross- sectional survey	A questionnaire was designed containing 39 items based on a systematic literature search and interviews with veterinarians. The 39-item scale was completed by a sample of 490 French veterinarians (average age = 37.34 years, SD = 13.39; 72.1% women, 27.9% men) together with the standardized measurement: MBI-GS (assessing burnout), SCL-90 (assessing the psychosomatic index) and three items measuring suicidal ideation. Statistical analyses (EFA and CFA (χ^2^ (669) = 1582.7; χ^2^/df = 2.36; CFI = 0.91; TLI = 0.90; RMSEA = 0.005) revealed eight factors corresponding to different aspects of the occupational stressors of veterinarians: negative work-home interactions, issues with coworkers, workload, responsibilities, financial issues, emotional demands, issues with clients and feeling of being in danger. The eight subscales show a satisfactory internal consistency (Cronbach’s alpha = 0.77 to 0.91) and criterion-related validity.
Andela, M. (2021); France [45]	Evaluation of the relationship between work-related stressors, burnout and suicidal ideation in veterinarians	IV	cross-sectional survey	The study was conducted with the same sample of 490 veterinarians as the 2020 study (average age = 37.34 years, SD = 13.39; 72.1% women, 27.9% men). Seven of the evaluated eight work-related stressors in this study were considered within the present study (workload (M = 29.88), emotional demands (M = 18.38), issues with clients (M = 30.43), issues with coworkers (M = 10.72), financial worries (M = 13.07), negative work–home interactions (M = 14.39) and having high responsibilities (M = 16.60)) and measured with the Veterinary Stressors Inventory (VSI). Burnout was evaluated with the Maslasch Burnout Inventory-General Survey (MBI-GS), and three items measured suicidal ideations (Cronbach’s alpha = 0.86). Statistical analysis indicated significant relationships between work-related stressors, burnout and suicidal ideation. Moreover, data demonstrated that burnout mediates the correlation between work-related stressors and suicidal ideation.
Batchelor et al. (2012); UK [6]	Ethical challenges and associated stress faced by veterinarian professionals was investigated via questionnaire	IV	cross-sectional survey	A brief questionnaire was sent to veterinarians in the UK. The questionnaire included demographic data as well as typical ethical challenges (e.g., euthanasia of a healthy animal, client financial constraints and client desire to continue treatment despite compromised animal welfare) to be assessed according to the stress triggered on a scale between 0 to 10. The survey was completed by 58 veterinarians (43 females and 15 males). 57% of the participants stated they face at least one to two ethical challenges per week. The clients desire to continue treatment despite compromised animal welfare was rated to be the most stressful challenge with a mean value of 9. Significant sex differences were found in stress ratings for healthy animal euthanasia (*p* = 0.02) and client wishing to continue treatment (*p* = 0.014) with females rating these challenges to be more stressful. The most common challenge faced was financial limitations restricting treatment options (55%).
Crane et al. (2015); Australia [35]	Investigation of morally challenging stressors in relation to psychological distress, perfectionism and resilience	IV	cross-sectional survey	An online survey was conducted of 540 Australian registered veterinarians (64.2% female; 35.8% male) aged 23 to 74 years (M = 41.06, SD = 11.53). The questionnaire included demographic variables and standardized measurements such as: the Multidimensional Perfectionism Scale, the Depression, Anxiety, Stress Scale, the Positive and Negative Affect Scale and the Brief Resilience Scale. Results indicated that suspected pet abuse was considered on average the most morally significant issue faced (M = 84.23, SD = 20.46). In addition, the data showed that working with clients who could not pay for recommended treatment was considered the least morally significant (M = 59.84, SD = 28.40) but most frequently endorsed stressor (n = 530), and that performing euthanasia was generally the most frequently occurring stressor (M = 7.41, SD = 1.54), nearly tied with the least morally significant stressor (M = 60.08, SD = 33.77). Statistical analyses moreover demonstrated that although morally significant stressors were associated with an increase in milder expressions of stress, they did not appear to be associated with more severe impairments in psychological well-being (*p* = 0.89). Rather, it was the combination of these triggering stressful events and trait perfectionism that appeared to create vulnerability to moral stressors (*p* = 0.97).
Griek et al. (2018); U.S. [36]	Development of a comprehensive taxonomy of practice-related stressors experienced by US veterinarians	IV	cross-sectional survey	A questionnaire was designed to evaluate the prevalence of risk factors for suicide, attitudes toward mental illness and practice-related stressors among 11,627 U.S. veterinarians. The questionnaire included demographic data and questions regarding attitudes toward mental health, history of depression and mental health treatment, stressors related to veterinary medicine and satisfaction related to veterinary medicine. In addition, veterinarians had the opportunity to openly identify individual work-related stressors. A subset of 1422 US veterinarians provided written responses to the questionnaire. Based on quantitative and qualitative results, a model was developed with 15 categories of general practice-related stressors and 40 subcategories of more specific practice-related stressors. The most common practice-related stressors included financial insecurity (20.3%), problems with clients (17.9%), problems with staff or interpersonal issues (12.7%) and work–life balance (11.7%). The most common subcategories were clients unwilling to pay (8.3%), low income (6.9%), cost of maintaining practice (3.9%) and government or state agency policies (3.4%).
Kassem et al. (2019); U.S. [28]	Analyzing attitudes toward mental illness among U.S. veterinarians	IV	cross-sectional survey	A questionnaire was developed to analyze characteristics (demographic, occupational and mental health) associated with negative attitudes toward mental illness among U.S. veterinarians. The questionnaire included demographic data, the Kessler Psychological Distress Scale and questions regarding suicidal ideation, attempted suicide, depression, treatment effectiveness and social support. A total of 9522 data sets were included in statistical analyses (69.2% female and 30.8% male). Results demonstrated that 47.3% of the sample had a negative attitude toward social support and 3.1% had a negative attitude toward treatment effectiveness. Specific demographic characteristics favored the negative attitude toward treatment effectiveness (male (40.9%), solo practitioners (27.2%), with evidence of serious psychological distress (22%) and those reporting suicidal ideation after graduating from veterinary school. Typical characteristics for a negative attitude toward social support were: female (75.2%), childless (46.8%), unmarried (26.5%), 40–50 years old (47.3%), solo practitioners (71.1%), with evidence of serious psychological distress, those reporting depression after graduating from veterinary school (24.1%).
Kipperman et al. (2017); U.S. and Canada [35]	Investigation of small animal veterinarians’ opinions and actions related to the cost of care and its effects on satisfaction and burnout	IV	cross-sectional survey	An online survey was conducted to identify small animal veterinarians’ opinions and actions related to the cost of care, barriers to client education about the cost of veterinary care and the impact of economic constraints on patient care and outcomes, as well as professional satisfaction and burnout. The questionnaire included demographical data, questions regarding the frequency with which practitioners discuss aspects of the treatment with animal caregivers, questions regarding the prevalence of professional burnout or career dissatisfaction, questions regarding the effect of pet owner awareness of veterinary care costs and questions regarding pet health insurance. In total, 1122 data sets were included in statistical analyses (68.5% female, 31.5% male; median number of years in practice = 15). Results showed that a majority of small animal veterinarians indicated that client economic limitations affected their ability to provide the desired care for the animals (57%). In addition, findings demonstrated that 49% of the sample reported a moderate-to-substantial level of burnout. Furthermore, only 31% and 23% of respondents routinely discussed veterinary costs and pet insurance, respectively, with clients before pets became ill, and lack of time was cited as a reason for forgoing those discussions.
Mastenbroek et al. (2014); The Netherlands [31]	Examining burnout, work engagement, and gender differences among veterinarians who graduated in the past 10 years	IV	cross-sectional survey	A self-report questionnaire was conducted to analyze levels of burnout and work engagement as well as characteristics predicting burnout and work engagement. The questionnaire included demographic data and the following standardized questionnaires: the Utrecht Work Engagement Scale, the Maslach Burnout Inventory-General Survey and the Veterinary Demands and Resources Questionnaire. In total, 860 data sets were included in the statistical analyses (73% females and 27% males; mean age = 32 years, SD = 4.4). Results overall demonstrated that the levels of exhaustion (M = 1.66, SD = 1.14), cynicism (M = 1.18, SD = 1.10) and work engagement (M = 3.63, SD = 1.05) were significantly lower among veterinarians compared to the norm group (d = 0.1 for all three measures). Moreover, data indicated that male veterinarians were less exhausted (B = −0.24, Beta = −0.09) and more engaged (B = 0.23, Beta = 0.10) than female veterinarians. In addition, results showed that female veterinarians experience slightly higher job demands and moderately lower professional and personal resources compared to their male counterparts. Furthermore, results suggest that variance in exhaustion and cynicism is best explained by job demands (Exhaustion: R2 (male) = 0.52, R2 (female) = 0.49; Cynicism: R2 (male) = 0.49, R2 (female) = 0.40) and job resources (Exhaustion: R2 (male) = 0.38; R2 (female) = 0.33; Cynicism: R2 (male) = 0.56; R2 (female) = 0.33) and to a lesser extent by personal resources (Exhaustion: R2 (male) = 0.21; R2 (female) = 0.19; Cynicism: R2 (male) = 0.10; R2 (female) = 0.19). Variance in work engagement may be best explained by job (R2 (male) = 0.44; R2 (female) = 0.48) and personal resources (R2 (male) = 0.42, R2 (female) = 0.31).
Perret et al. (2020); Canada [24]	Investigation of the association between veterinarian mental health and veterinary client satisfaction	IV	cross-sectional survey	In order to evaluate the association between veterinarian mental health and veterinary client satisfaction, Canadian veterinarians were surveyed online from 2017 to 2019 using an online questionnaire. The survey included demographic data, career related questions and the following standardized measurements: he Perceived Stress Scale, the Maslach Burnout Inventory, the Hospital Anxiety and Depression Scale, the Professional Quality of Life Scale and the Connor-Davidson Resilience Scale. In addition, clients were also surveyed separately using the Client Satisfaction Questionnaire. Overall data sets of 60 veterinarians (65% females and 35% males) and 995 clients (73.3% females and 26.2% males) were included in statistical analyses. Results demonstrated non-linear associations between client satisfaction and veterinarian mental health measures indicating that a higher client satisfaction might be associated with a lower mental health status among veterinarians.
Platt et al. (2012); UK [22]	Obtaining information from veterinarians with suicidal ideation or behavior about factors associated with suicidality in their profession	/	mixed-methods design	To investigate work-related factors associated with suicidality among veterinarians 21 UK veterinarians (76% females, 24% males) who had attempted suicide or reported recent suicidal ideation were interviewed using semi-structured interview schedules. The interviews included topics relating to contributory factors, coping mechanisms and suicide prevention in the veterinary profession. In addition to the interviews some topics were also assessed in part by fixed-response scales such as the Suicidal Intent Scale or items from the Motives for Parasuicide Questionnaire. Results demonstrated common factors contributing to suicidal behavior were workplace relationships, career concerns, problems with patients, number of hours and volume of work and responsibilities. Although 2/3 of participants reported concurrent difficult life events about half of the veterinarians had received a psychiatric diagnosis after their suicidal behavior. Participants suggested several possible preventive measures.
Platt et al. (2012); UK [41]	Investigation of suicidal behavior and psychosocial problems in veterinary profession	II	Systematic review	A systematic review was performed to summarize the knowledge on suicidal behavior and psychosocial problems in the veterinarian profession. Data from 52 studies (from 48 papers) concerning suicidal behavior, mental health problems, stress, burnout, occupational difficulties and psychosocial characteristics of veterinarians were analyzed. Results demonstrated that a majority of studies dealt with occupational stressors among veterinarians including managerial aspects of the job, long working hours, heavy workload, poor work–life balance, difficult client relations and performing euthanasia. In addition, some studies suggested that young and female veterinarians are at higher risk for negative outcomes such as suicidal thoughts, psychological problems and job dissatisfaction.
Pohl et al. (2022); Germany [20]	Investigation of work-related stressors and their effects on mental health among veterinarians	II	Systematic review	A systematic review was performed to provide an overview of existing work-related stressors and their effects on mental health among veterinarians. Therefore, 21 studies related to work-related stressors, psychological workload and potential effects such as burnout or depression were analyzed. Results demonstrated a high prevalence of psychological stressors in veterinary practice. Studies in this context indicated that the risk of burnout, anxiety and depressive disorders are higher among veterinarians than in the general population. In addition, the results showed that working hours and ethical dilemmas are the main sources of stress perceived as more stressful by female veterinarians than by their male colleagues.
Väärikkälä et al. (2020); Finland [46]	Evaluation of job satisfaction among veterinarians working in the field of animal welfare control	IV	cross-sectional survey	An online questionnaire was conducted to evaluate working conditions and well-being at work of Finnish veterinarians. The questionnaire included demographic data, questions regarding work experiences, current position, work content, positive features as well as challenges at work, job satisfaction and negative side effects of work, work-related stressors, social support and use of enforcement measures. In total, 73 responses of veterinarians working in the field of animal welfare control were included in statistical analyses. Results demonstrated that more than a half of respondents reported work-related stress caused by factors such as Threatening situations (r = 0.37, *p* = 0.01), disturbed work–private life balance (r = −0.71, *p* < 0.001) and a high amount of overtime work (r = 0.44, *p* = 0.01) were found to be frequent underlying causes of stress. In addition, data indicated that the more veterinarians worked over time and participated in animal welfare control outside office hours, the more often they perceived the commitment to work as too high (r = 0.55 and r = 0.31, respectively, *p* < 0.01 for both). No direct relationship between work support and work–private life balance was found, but data showed that support might help decreasing sleeping disorders (r = 0.31, *p* = 0.01).
Wallace (2017); U.S. [43]	Evaluation of work-related stressors, burnout and suicidal ideations among veterinarians	IV	cross-sectional survey	An online questionnaire was conducted to investigate links between work-related stressors, burnout and suicidal ideation among U.S. veterinarians. In addition, individual coping strategies were explored. The questionnaire included demographic variables as well as the following standardized measurements: the National Survey of Psychiatric Morbidity and the Maslach Burnout Inventory. Additionally, questions regarding job demands, job control, social support and coping strategies were asked. In total, data sets of 464 veterinarians working in clinical practice were included in statistical analyses (31% females and 69% males; mean age = 43 years). Results demonstrated that burnout was associated with suicidal thoughts (Beta = 0.37) and appeared to be particularly important in understanding how unrealistic client demands indirectly contribute to suicidal ideation (Beta = 0.12). Furthermore, data indicated that job demands (especially work overload) (Beta = 0.25) are relevant indicators to burnout and that working conditions that are emotionally exhausting for veterinarians might foster suicidal ideation (work overload (r = −0.15), euthanasia (r = −0.40), animal suffering (r = −0.23), unrealistic clients (r = −0.04). Results also showed that emotional avoidance strategies and alcohol abuse not only exacerbate burnout (Beta(avoidance) = 0.11; Beta(alcohol) = 0.12) and suicidal thoughts (Beta(avoidance) = 0.06; Beta(alcohol) = 0.13), but also increase the harmful effects of certain job demands (β = 0.13). Social support (Beta(Burnout) = −0.16; Beta(Suicide) = −0.13) and a supportive work environment (β = −0.13) has proven to be a relevant coping resource.

**Table 4:** Psychological consequences.

First Author, Year of Publication and Location	Subject	OCEBM Level (2011)	Study Type/Design	Main Findings
Best et al. (2020); Canada [48]	Determination of depression, anxiety, compassion fatigue, burnout and resilience among Canadian veterinarians	IV	cross-sectional survey	An online questionnaire was conducted to evaluate the prevalence of depression, anxiety, compassion fatigue, burnout and resilience in veterinarians in Ontario, Canada. The questionnaire included demographic data as well as the following standardized measurements: the Hospital Anxiety and Depression Scale, the Maslach Burnout Inventory-Human Services Scale, the Professional Quality of Life scale and the Connor-Davidson Resilience Scale. Data were collected from 412 Canadian veterinarians (255 females (69.5%), 113 males (30.5%); mean age = 45 years, SD = 11.8). Statistical analyses showed that participants in the present study had poorer mental health compared with the general population (e.g., Depression (M = 5.2), Anxiety (M = 8.8), Burnout (M = 25.9)). In addition, the data showed that female veterinarians tended to have poorer mental health compared to male veterinarians.
Crane et al. (2017); Australia [49]	Investigation of the association between perceived skill-transferability and suicide-related behavior and cognitions among veterinarians	III	longitudinal study	An online survey was conducted and sent to Australian veterinarians at two different times within a period of 12 months. The questionnaire included demographic data and the following standardized measurements: the Suicidal Behaviors Questionnaire-Revised, The Intention to Leave Scale and the Depression, Anxiety, Stress Scale. To the T1 survey 462 veterinarians responded. Of these respondents 161 veterinarians had usable data across both time periods (65.2% females and 34.8% males). Results demonstrated that at T1 24.80% of veterinarians were in the high suicide-related cognitions and behavior category, and at T2, this number increased to 26.10%. In addition, data indicated a significant effect of T1 depression levels on the prediction of the overall-probability of suicide-related cognitions and behavior (*p* < 0.001). Findings also showed that overall, reported suicide-related behavior and cognitions increased within the 12 months, when there was a high intention to leave in T1 but only when skill transferability to other professions were perceived to be limited (*p* = 0.04)
Neill et al. (2022); U.S. [50]	Examining burnout in the veterinary profession with a focus on financial reasons.	IV	cross-sectional survey	An online survey was conducted to analyze burnout prevalence, turnover and reduced working hours in U.S. veterinarians from 2016 to 2020. The questionnaire included demographic data, questions about veterinary education, employment, compensation, debt, well-being and a standardized measure to assess burnout (the Professional Quality of Life Scale). The level of burnout was used to calculate conditional probabilities on turnover and reduced working hours due to burnout. In total, data from 5786 associate veterinarians in private practice were used for statistical analyses. Results demonstrated that that the economic cost of burnout due to turnover and reduced working hours is ~$997 million in lost revenue. The cost of employee turnover is almost twice as high as the cost of lost productivity due to reduced working hours.
Nett et al. (2015); U.S. [47]	Investigation of risk factors for suicide, mental-illness and work-related stressors among veterinarians	IV	cross-sectional survey	An online questionnaire was conducted to evaluate the prevalence of suicide risk factors, attitudes toward mental illness and practice-related stressors among U.S. veterinarians. The survey included demographic data, practice setting, history of depression and mental health treatment, attitudes toward mental illness and mental health treatment, stressors related to veterinary practice and satisfaction related to practicing veterinary medicine. Additionally, Kesslers Psychological Distress Scale was used to measure the presence of serious mental illness. A total of 11,627 respondents were included in statistical analyses (69% females and 31% males). Results demonstrated that 31% veterinarians experienced depressive episodes, 17% experienced suicidal ideation and 1% attempted suicide after leaving veterinary school. Nine percent of respondents showed serious psychological distress at the time of the survey. Only 32% of participating veterinarians strongly agreed that people are sympathetic toward persons with mental illness. The most frequently reported practice-related stressor was the demands of practice.
Perret et al. (2020); Canada [24]	Investigation of perceived stress, burnout, depression, anxiety, compassion fatigue, compassion satisfaction, resilience and suicidal ideation among Canadian veterinarians	IV	cross-sectional survey	An online survey was conducted to evaluate perceived stress, burnout, depression, anxiety, compassion fatigue and resilience in Canadian veterinarians. The questionnaire included demographic data as well as the following standardized measurements: the Perceived Stress Scale, the Maslach Burnout Inventory, the Hospital Anxiety and Depression Scale, the Professional Quality of Life Scale and the Connor-Davidson Resilience Scale. In addition, questions regarding suicidal ideation, regarding satisfaction with available support and regarding the mental health history were asked. In total, 1403 data sets were included in statistical analyses (76.4% females and 23.6% males). Overall results showed that respondents had significantly higher mean scores for subscales of burnout and compassion fatigue, anxiety and depression and significantly lower mean resilience. In addition, data demonstrated that female veterinarians had significantly higher mean scores for perceived stress (PSS: M(arithmetic) = 17.6, M(geometric) = 16.0, SD = 7.0; *p* < 0.001), emotional exhaustion (MBI: M(arithmetic) = 27.2, M(geometric) = 23.1, SD = 13.2; *p* < 0.001), burnout (ProQOL: M(arithmetic) = 25.5, M(geometric) = 24.5, SD = 7.0; *p* < 0.001), secondary traumatic stress (ProQOL: M(arithmetic) = 24.0, M(geometric) = 23.1, SD = 6.4; *p* < 0.001, anxiety (HADS: M(arithmetic) = 8.7, M(geometric) = 7.5, SD = 4.2; *p* < 0.001) and depression (HADS: M(arithmetic) = 5.2, M(geometric) = 4.2, SD = 3.7; *p* < 0.001) and significantly lower mean resilience in comparison with their male colleagues. Moreover, results indicated that the 12-month prevalence of suicidal ideation among participants was 26.2%, which was much higher than the estimated prevalence in the international general population (2.1% to 10.0%; *p* < 0.001).

## Data Availability

Not applicable.
